# Directional flow of brain connections and neurodevelopmental outcomes in healthy full-term newborns

**DOI:** 10.1016/j.neulet.2025.138371

**Published:** 2025-09-03

**Authors:** Venkata Chaitanya Chirumamilla, Sarah B. Mulkey, Tayyba Anwar, Robin Baker, G.Larry Maxwell, Josepheen De Asis-Cruz, Kushal Kapse, Catherine Limperopoulos, Adre du Plessis, R.B. Govindan

**Affiliations:** a The Zickler Family Prenatal Pediatrics Institute, Children’s National Hospital, Washington, DC, United States; b Department of Pediatrics, The George Washington University School of Medicine and Health Sciences, Washington, DC, United States; c Department of Neurology, The George Washington University School of Medicine and Health Sciences, Washington, DC, United States; d Department of Neurology, Children’s National Hospital, Washington, DC, United States; e Inova Women’s and Children’s Hospital, Fairfax, VA, United States; f Fairfax Neonatal Associates, Fairfax, VA, United States; g Developing Brain Institute, Children’s National Hospital, Washington, DC, United States; h Division of Diagnostic Imaging and Radiology, Children’s National Hospital, Washington, DC, United States

**Keywords:** Directional flow, HD-EEG source analysis, Linearly constrained minimum variance, beamformer, Neurodevelopmental outcome

## Abstract

**Objective:**

We examined whether directional flow among brain hubs in healthy-term infants is associated with neurodevelopmental outcomes at two years of age.

**Methods:**

High-density electroencephalography (EEG) was collected within 72 h after birth. Neurodevelopmental outcomes (cognitive, language, and motor scores) were measured using Bayley Scales of Infant Development-III (BSID-III) at two years. Source signals were extracted from the hubs, and directed information flow from hub was calculated using partial directed coherence method in delta band. The relationship between information flow and BSID-III scores was assessed using stepwise regression.

**Results:**

Forty-seven newborns had EEG and BSID-III scores. Efferent flow from the left amygdala (t-statistic = −2.97, p = 0.027), right amygdala (t-statistic = −2.15, p = 0.03), and right caudate nucleus (t-statistic = −2.16, p = 0.036) were negatively associated, while the left pallidum (t-statistic = 2.72, p = 0.02) was positively associated with cognitive scores. The efferent flow from the right amygdala (t-statistic = −2.34, p = 0.03) was negatively associated with language scores, while efferent flow from the brainstem (t-statistic = 2.38, p = 0.03) was positively associated with motor scores.

**Conclusions:**

Efferent output from specific hubs at birth is associated with neurodevelopmental outcomes at two years of age.

## 1. Introduction

Early brain development is crucial for later cognitive, language, motor, and socio-emotional skills. Understanding neural communication in healthy full-term newborns may help identify early markers of neurodevelopmental risk. Electroencephalography (EEG) signals have far better temporal resolution than functional magnetic resonance image (fMRI) signals, and their easy application allows for study of neurodevelopment at the bedside. Due to its excellent spatial resolution, fMRI Blood oxygenation level-dependent (BOLD) signals are widely used to study brain functional networks [1]. The functional brain connectivity metrics of term-born individuals studied in the first weeks of life have served as a benchmark to identify atypical patterns of preterm infants, which are related to the development of impaired neurodevelopment [2]. The resting state networks identified using the functional connectivity of preterm infants showed impairment in the functional connectivity depending on their age at birth. Furthermore, the preterm infants showed hyper connectivity in the superior parietal lobules that has been related to the developmental coordination disorder [3]. The functional connectivity metrics thus offer a reliable tool to study maturational changes in the brain.

High-density EEG (HD-EEG) offers high temporal resolution in the order of milliseconds, making it well suited to study fast neural dynamics [4,5]. However, EEG does not have spatial resolution to study the connectivity in the subcortical regions. Combining EEG with advanced analytical tools makes it possible to derive source signals that are equivalent to fMRI BOLD signals [6]. We and others have demonstrated the feasibility of performing functional brain network analysis using HD-EEG data [7–9]. Our previous study demonstrated that the functional brain connectivity network in term low-risk newborns exhibits highly connected brain regions (hubs), and there is a high degree of communication among hubs, and these results agreed with those from fMRI studies [10,11].

Although the functional connectivity metrics identify the network architecture, they do not provide the directional flow of information [12]. The directionality of connectivity—indicating the flow of information between brain regions—may provide valuable insights into neural development that are not revealed by undirected measures. For instance, efferent (outgoing) and afferent (incoming) information flows can reflect distinct functional roles of brain regions in supporting cognitive and behavioral maturation. In our recent study, we performed directed flow analysis in the delta frequency band among hubs, which showed that the brainstem had a higher afferent flow, and the left putamen showed a lower afferent flow [13]. Furthermore, the right pallidum had a higher efferent flow, and the brainstem had a lower efferent flow. Despite growing interest in neonatal brain connectivity, it remains unclear whether early directional connectivity metrics are predictive of long-term neurodevelopmental outcomes. Demonstrating such a relationship would establish the clinical value of directionality as a complementary or possibly more informative predictor compared to conventional undirected metrics.

To address this gap, the present study investigates the association between directed information flow among hubs in the neonatal brain and neurodevelopmental outcomes at two years of age. The source EEG signals were extracted from hubs, and the directional flow among them was characterized in the delta frequency band using the partial directed coherence (PDC) method, which is a frequency-domain representation of Granger causality analysis technique. Neurodevelopmental outcomes at age two years were evaluated using the Bayley Scales of Infant and Toddler Development-III (BSID-III). Based on our previous findings that spectral power and functional connectivity immediately after birth were linked to neurodevelopmental outcomes at two years of age [6,14], we hypothesized that directed information flow among hubs immediately after birth would also be associated with later neurodevelopmental outcomes.

## 2. Materials and methods

### 2.1. Participants description

We recruited low-risk term infants (37 to 41 weeks gestation) at Inova Women’s Hospital, Fairfax, Virginia, between May 2017 to June 2018, within 72 h after birth. The Institutional Review Boards of Children’s National Hospital, Washington, DC, and Inova Fairfax Hospital, Falls Church, Virginia, approved the study (IRB Number: 16–2559, approved 03/27/2017) and informed consent was obtained in all cases. The inclusion criteria were birth weight between the 10th and 90th percentile for gestational age and singleton pregnancies without complications. Infants with genetic or metabolic syndromes and previously diagnosed maternal diabetes, substance abuse, or hypertension were excluded.

### 2.2. EEG signal acquisition and preprocessing

All EEG recordings were performed using the 124-channel Hydrocel Geodesic Sensor Net System (Electrical Geodesics, Inc., Eugene, OR, USA). The infants were placed supine in a bassinet, and an EEG net was applied. All electrode impedances were checked and kept appropriate before the start of each recording, and raw data were sampled at either 250 Hz or 1000 Hz. The ECG (electrocardiogram) and video signals were simultaneously recorded along with the EEG. EEG data recorded at 1000 Hz were down-sampled to 250 Hz, and a fourth-order Butterworth filter was applied with a cutoff frequency of 0.1 Hz. All EEG signals were re-referenced to the average of all channels using a frequency domain approach[15].The volume conduction and ECG interference in EEG data were attenuated using the frequency-based null-coherence method [15]. An experienced neurophysiologist used the EEG and simultaneously recorded video data and classified EEG data into one of the four categories: active sleep, quiet sleep, awake, and artifact. The artifacts were identified based on visual inspection in half of the studies. An algorithm was developed to identify the artifacts in the remaining half of the studies. The EEG data was segmented into 1-minute intervals, and the standard deviation was computed for each channel within each segment. ROC analysis showed that a 25 µV per-channel threshold could reliably distinguish artifacts from sleep-wake patterns (AUC > 0.8). Segments with any channel exceeding this threshold were excluded from further analysis. EEG recordings lasted approximately 50 to 60 min. For each subject, the first continuous 3-minute segment of artifact-free data was selected based on the criteria above. Due to the high prevalence of active sleep in the cohort, 96 % of the selected segments corresponded to active sleep.

### 2.3. Source activity reconstruction using linearly constrained minimum variance (LCMV) beamformer

Source analysis was performed using Fieldtrip functions [16,17] and custom Matrix Laboratory (MATLAB) functions. The LCMV beamformer was used to extract the source time series from the selected artifact-free EEG data. This method calculates a spatial filter that relates the electrical activity measured using EEG sensors to the neural activity inside the brain. The head model was constructed using the T2-weighted MRI of a full-term newborn (GA 41 weeks). The MR images were transformed into MNI (Montreal Neurological Institute) space and segmented into brain, skull, and scalp using the FMRIB Software Library-Brain Extraction Tool (FSL-BET) (FSL, V6.0) [18]. The conductivities of the brain, skull, and scalp were set to 1.79 S/m, 0.2 S/m, and 0.43 S/m, respectively [19]. The medoid voxel was computed for each of the 93 brain regions specified by the pediatric automated anatomical labeling (AAL) atlas, and the source time course was calculated [6]. The functional connectivity between brain regions was calculated separately for every subject in the delta band ([0.5 4 Hz]) [7]. Individual matrices were averaged to obtain the group-level matrix. Connectivity strength per region was computed by summing across matrix rows. Regions exceeding the mean plus one standard deviation were classified as hubs, and information flow among them was calculated in the delta band using the partial directed coherence (PDC) method [20]. The PDC analysis was confined to the delta frequency band as the power in this frequency band is dominant in the newborn EEG [21].

### 2.4. Estimating the directional flow using PDC

The directional flow among hubs was estimated using PDC method developed by Baccala and Sameshima [22], applied to source-level EEG signals extracted from hub regions.. Let $x\left(n\right)$ be the matrix of time series from N different hubs, which can be represented using the vector autoregressive model of order p as shown in equation (1):

(1) $$x\left(n\right)=\sum_{r=1}^{p}A\left(r\right)x\left(n-r\right)+\varepsilon \left(n\right)$$

Where $A\left(r\right)$ is the matrix of the autoregressive coefficients, *p* is the model order calculated using the Akaike Information Criterion (AIC) and set to 50 in our analysis, and $\varepsilon \left(n\right)$ is the multivariate gaussian white noise process.

The PDC from $x_{b}$ to $x_{a}$ is calculated using the formula shown in equation (2)

(2) $$\pi_{ab}\left(f\right)=\frac{A_{ab}\left(f\right)}{\sqrt{{v_{b}}^{\tau }\left(f\right)\cdot v_{b}\left(f\right)}}$$

Where $A\left(f\right)$ is the transformation of $A\left(r\right)$ into frequency domain, $v_{b}\left(f\right)$ represents the column b of the $A\left(f\right)$ and τ represents the complex conjugate operator.

The calculated PDC was considered as significant if it exceeded the value calculated using equation (3)[23].

(3) $${\left(\frac{{\hat{C}}_{ab}\left(f\right)\chi_{1,1-a}^{2}}{N\sum_{k}{\left|A_{kb}\left(f\right)\right|}^{2}}\right)}^{1/2}$$

with $\hat{C_{ab}}\left(f\right)=\sum_{aa}\left[\sum_{k,l=1}^{p}H_{bb}\left(k,l\right)\left(\cos\left(kf\right)\cos\left(lf\right)+\sin\left(kf\right)\sin\left(lf\right)\right.\right]$ in which $H_{bb}\left(k,l\right)$ is the elements in the inverse covariance matrix of the vector autoregressive process of x. $\chi_{1,1-a}^{2}$ denotes the 1−α quantile of the $\chi^{2}$ distribution with one degree of freedom. The efferent flow from a particular hub was calculated as the sum of directional flow from that hub to all other remaining hubs.

### 2.5. Neurodevelopment outcome measurements

Neurodevelopmental outcomes were assessed at two years of age by a certified developmental therapist using the BSID-III. Composite scores for cognitive, language, motor, and socio-emotional domains were included in the analysis [24].

### 2.6. Statistical analysis

The continuous data were shown as means (standard deviation) and categorical data as counts (percentage). We followed the convenient sampling approach in this retrospective analysis of the prospectively collected data. We used the available data to explore the relationship between information flow among hubs quantified using PDC and developmental outcomes. Stepwise multiple linear regression model was used to identify the brain regions in which information outflow was related to neurodevelopmental outcomes (cognitive, language, and motor) at two years of age. Gestational age (GA) at birth was included as a covariate in all regression models to account for its potential confounding effect. The p-values obtained from the regression analysis were corrected for multiple comparison using the Benjamini-Hochberg approach at α=0.05.

## 3. Results

One hundred sixty-three term newborns were enrolled in this study. Sixty-seven (41 %) had neurodevelopmental evaluation with the BSID-III at two years of age. This low follow-up rate was largely due to the COVID-19 pandemic. Of these 67 newborns, 47 had continuous artifact-free EEG for 3 min. The clinical and demographic data of the studied participants are in Table 1. More than 96 % of the newborns were in the active sleep state and the rest were in an awake state during their EEG.

**Fig. 1.**
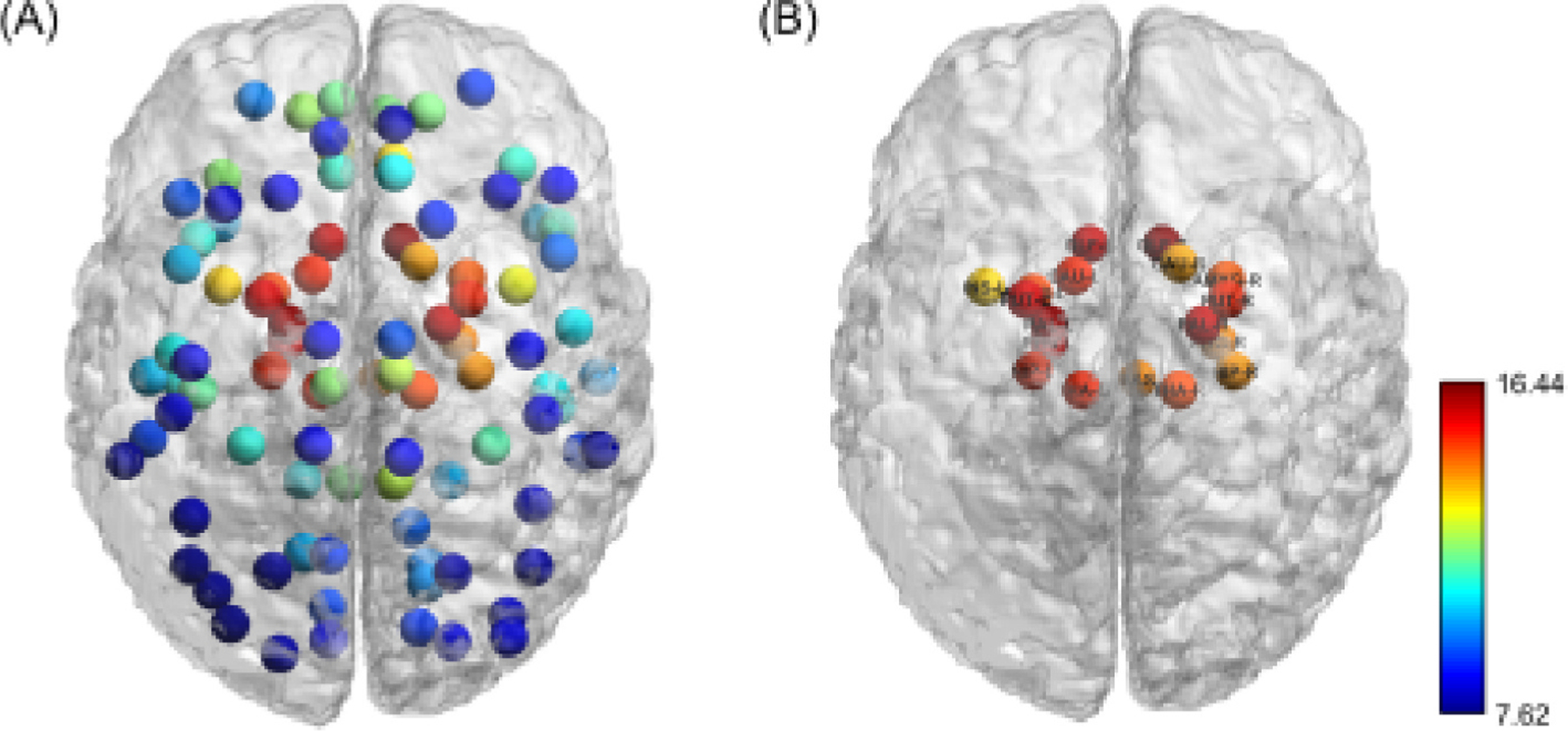
Group-level brain connectivity strength and identified network hubs. (a) Group-level connectivity strength across all brain regions, averaged across subjects. Warmer colors indicate regions with higher connectivity strength. (b) Regions identified as network hubs based on connectivity strength exceeding 1 standard deviation above the mean. A total of 18 hubs were identified, including bilateral olfactory cortex (OLF-L/R), hippocampus (HIP-L/R), parahippocampal gyrus (PHG-L/R), amygdala (AMYG-L/R), caudate (CAU-L/R), putamen (PUT-L/R), pallidum (PAL-L/R), thalamus (THA-L/R), insula (INS-L), and the brainstem (BAS). L = left hemisphere; R = right hemisphere.

To provide a comprehensive overview of the functional brain network, we calculated group-level connectivity strength across all brain regions (Fig. 1a). Using a threshold of 1 standard deviation above the mean, we identified 18 network hubs, including bilateral olfactory cortex, insula, hippocampus, parahippocampal gyrus, amygdala, caudate, putamen, pallidum, thalamus, and brainstem. Their spatial distribution is shown in Fig. 1b. We assessed the consistency of these hubs at the individual-subject level and found that subcortical regions such as the olfactory cortex, pallidum, parahippocampal gyrus, putamen, thalamus, and hippocampus were present in 67–94 % of subjects, whereas regions such as the insula and caudate were present in only 42–55 %. Based on these results, we identified these regions as representative hubs for group-level analysis.

Stepwise regression analysis revealed that neurodevelopmental outcomes at two years of age were significantly associated with directional flow among hubs immediately after birth (Table 2). The efferent flow from the left amygdala (t-statistic = −2.97, p = 0.027), right amygdala (t-statistic = −2.15, p = 0.0367), and right caudate (t-statistic = −2.16, p = 0.036) were negatively associated with cognitive score, while efferent flow from the left pallidum (t-statistic = 2.72, p = 0.02) was positively related to cognitive score. The efferent flow from the right amygdala (t-statistic = −2.34, p = 0.03) was negatively associated with language score, while the efferent flow from the brainstem (t-statistic = 2.38, p = 0.03) was positively related to motor score in the delta band (p < 0.05).

## 4. Discussion

In this study, we describe the association between the directional flow among hubs in the newborn brain functional network and neurodevelopmental outcomes at two years of age. A higher efferent flow from the left amygdala, right amygdala and right caudate was associated with lower cognitive scores on the BSID-III. In contrast, a higher efferent flow from the left pallidum was associated with higher cognitive scores. A higher efferent flow from the right amygdala was associated with lower language scores. A higher efferent flow from the brainstem was associated with higher motor scores. These findings provide new insights into early neurodevelopmental markers and highlight the critical role of specific brain regions in neurodevelopmental outcomes.

The amygdala is well developed at birth and plays an essential role in processing emotional responses, which are critical during early postnatal development [25,26]. It also projects to the cortical regions, including the parietal, occipital, and temporal regions, involved in emotion regulation and attention [27]. Our results showed that increased efferent flow from amygdala is negatively associated with cognitive outcomes, suggesting that higher activity in this region may disrupt typical development. This aligns with prior study showing larger amygdala volume in infants who later develop autism spectrum disorder (ASD) [28]. Additionally, caudate volume at 12 months has been positively associated with repetitive behaviors at two years of age in infants with fragile X syndrome [28]. Thus, aberrant higher efferent flow from the amygdala and caudate identified in our cohort, may indicate an atypical connectivity pattern, warranting further research studies to investigate its potential as a biomarker for neurodevelopmental impairments.

The association between efferent flow in pallidum and cognitive outcomes provides additional insights into early brain development. Previous research study on ex-preterm infants showed that infants with higher pallidum connectivity at term-equivalent age had better cognitive outcome at two years of age[29]. Similarly, we found efferent flow of the left pallidum was positively associated with cognitive scores. This suggests that pallidum may play a critical role in facilitating early cognitive development. By identifying directional flow, our findings underscore the importance of both structural and functional connectivity in this region.

The association between brainstem efferent flow and motor outcomes highlights the brainstem’s foundational role in motor development. The brainstem is known to regulate autonomic and motor functions in newborns [30,31], and our results emphasize its contribution to motor skill acquisition. The positive association between efferent flow from the brainstem and motor outcome suggests that directional connectivity from this region facilitates motor system maturation.

A previous study showed that larger right amygdala volume at six months was associated with lower expressive and receptive language scores at 2, 3, and 4 years [32]. Consistent with this, we observed that infants with higher efferent flow from the right amygdala had lower language scores at two years of age. These results highlight the right amygdala’s critical role in early language development and its potential as a biomarker for identifying individuals with language delays.

Compared to our previous analysis using undirected connectivity [14], the directional flow analysis offers a more mechanistic view of early brain network dynamics. While undirected metrics reflect regional synchrony, directed connectivity identifies source regions of neural communication. For instance, increased efferent flow from the amygdala and caudate, linked to poorer cognitive and language outcomes, suggests early overactivation from these hubs may disrupt neurodevelopment. These insights were not captured by undirected measures, highlighting the added value of effective connectivity metrics.

This study has several strengths, such as EEG recordings from newborns immediately after birth and the application of PDC method to quantify directional flow in the delta band from source-reconstructed EEG data. Due to the computationally intensive nature of the PDC calculations, we limited directional flow analysis to the identified hub regions. Future work using more scalable methods may help capture directed connectivity across the full network. While our current analysis focused on a static time window, applying time-varying PDC in future studies could better capture dynamic changes in connectivity over time [33].

In conclusion, our study described a significant association between directed information flow among hubs at birth and later neurodevelopmental outcomes. Our findings link specific patterns of information flow to cognitive, language, and motor scores, establishing a foundation for future research that will focus on early detection and intervention in at-risk infants.

## 6. Significance statement

The direction of communication among the highly connected brain regions in term low-risk newborns may serve as a biomarker to identify newborns at risk for poor neurodevelopmental outcomes.

## Figures and Tables

**Table 1:** The demographic and clinical characteristics of 47 term newborns included in this study.

Characteristics	Data
Postnatal age at EEG study in days, mean (SD)	1.6 (0.6)
Birth gestational age in weeks, mean (SD)	39.2 (0.8)
Females, n (%)	25 (53 %)
Apgar score at 1 min, median (range)	8 (7–9)
Apgar score at 5 min, median (range)	9 (8–9)
**Delivery mode**	
Vaginal, n (%)	25 (53 %)
Cesarean section, n (%)	22 (47 %)
**BSID-III composite score, mean (SD)**	
Cognitive	106.4 (15.2)
Language	109.4 (15.8)
Motor	103 (12.3)

EEG: Electroencephalography, SD: standard deviation

**Table 2:** Efferent flow correlations between brain regions and BSID-III cognitive, language, and motor scores. The statistics are represented as t-statistic (p value, adjusted p value). The adjusted p values were obtained using the Benjamini-Hochberg approach with false discovery rate of 0.05.

Brain region	Cognitive score	Language score	Motor score
Right amygdala	−2.15 (0.036, 0.0367*)	−2.34(0.023, 0.03*)	–
Left amygdala	−2.97 (0.004, 0.027*)	–	–
Right Caudate	−2.16 (0.035, 0.036*)	–	–
Left Pallidum	2.72 (0.009, 0.02*)	–	–
Brainstem	–	–	2.38 (0.02, 0.03*)

BSID-III: Bayley Scales of Infant and Toddler Development, Third Edition

*: Adjusted p value.

## Data Availability

Data will be made available on request.
